# Prevalence and Correlates of Dyslipidemia Among Men and Women in Palau: Findings of the Palau STEPS Survey 2011–2013

**DOI:** 10.2188/jea.JE20170127

**Published:** 2019-03-05

**Authors:** Meishan Cui, Chifa Chiang, Renzhe Cui, Kaori Honjo, Hiroshi Yatsuya, Berry Moon Watson, Edolem Ikerdeu, Takashi Mita, Sherilynn Madraisau, Atsuko Aoyama, Hiroyasu Iso

**Affiliations:** 1 Public Health, Department of Social Medicine, Osaka University Graduate School of Medicine, Osaka, Japan; 2 Department of Public Health and Health Systems, Nagoya University School of Medicine, Aichi, Japan; 3 Psychology and Behavioral Sciences, Faculty of Medicine, Osaka Medical College, Osaka, Japan; 4 Department of Public Health, Fujita Health University School of Medicine, Aichi, Japan; 5 Ministry of Health, Republic of Palau; 6 Institute for Academic Initiatives, Osaka University, Osaka, Japan

**Keywords:** dyslipidemia, cholesterol, triglycerides, body mass index, Palau

## Abstract

**Background:**

Epidemiological evidence of dyslipidemia in Pacific Island countries is limited despite the knowledge that non-communicable diseases have a high burden in the region. We aimed to examine the prevalence and correlates of dyslipidemia among residents of Palau.

**Methods:**

The Palau STEPwise approach to Surveillance (STEPS), which was conducted from 2011 through 2013, comprised three parts: behavioral risk factors; physical measurements; and biochemical tests, covering areas such as blood lipids. We used STEPS-generated data to perform a cross-sectional study of 2,184 randomly selected Palau residents, comprising Palauans and non-Palauans aged 25–64 years.

**Results:**

The age-adjusted mean BMI was 29.3 kg/m^2^ in men and 29.9 kg/m^2^ in women; age-adjusted mean triglycerides value was 182 mg/dL in men and 166 mg/dL in women; and age-adjusted mean cholesterol was 178 mg/dL in men and 183 mg/dL in women. The prevalence of overweight/obesity (BMI ≥25 kg/m^2^) was 75% in men and 76% in women, and those of hypertriglyceridemia (triglycerides ≥150 mg/dL) and hypercholesterolemia (total cholesterol ≥200 mg/dL) were 48% in men and 41% in women and 18% in men and 23% in women, respectively. Mean values of total cholesterol were 177 mg/dL in Palauan men and 182 mg/dL in non-Palauan men. Mean values of triglycerides were 171 mg/dL in Palauan women and 150 mg/dL in non-Palauan women. Women living in rural areas showed a higher mean value of total cholesterol than those in urban areas.

**Conclusion:**

We found a high mean BMI and high prevalence of overweight/obesity and hypertriglyceridemia, but low mean total cholesterol and a low prevalence of hypercholesterolemia in Palau. Lipid profiles varied by age, ethnicity, and living area.

## INTRODUCTION

The Republic of Palau, a small island country in the Western Pacific Ocean, with a population around 20,000 as of 2005,^1^ suffers a high burden from non-communicable diseases (NCDs). According to the National Health Profile 2013 in Palau,^2^ the leading causes of deaths are cardiovascular disease (29.6%), cancer (17.9%), injury (13.0%), and diabetes (11.7%). Cardiovascular diseases have consistently been a leading cause of death in the country over the last 10 years. The Ministry of Health, Republic of Palau collaborated with the World Health Organization (WHO) and conducted nationwide NCD risk factor surveillance from 2011 through 2013. The WHO STEPwise approach to Surveillance (STEPS) is a simple, standardized method for monitoring NCD risk factors in WHO member states. By using the WHO STEPS standardized questionnaire and protocols, member states can not only monitor within-country trends, but also make comparisons across countries. STEPS offers an entry point for conducting chronic disease surveillance of low- and middle-income countries.^3^^,^^4^

According to a WHO report,^5^ ischemic heart disease is the leading cause of death globally, and many studies in Western and Asian countries have reported positive associations between dyslipidemia and risk of ischemic heart disease.^6^^–^^9^ However, few epidemiological studies have reported on the status of dyslipidemia in Pacific Island countries, which include Palau. Therefore, in the present study, we examined the prevalence of high total cholesterol and high triglycerides, as well as population mean levels of these lipids, and their correlates among adults in Palau. Because of a high burden of obesity in Palau, we hypothesized that Palau also has a high burden of dyslipidemia. For the correlates of dyslipidemia, we hypothesized that older people have higher values of blood lipids, that Palauans have higher values of blood lipids than non-Palauans, that rural residents have higher prevalence of dyslipidemia than urban residents, and that people with lower levels of education have higher prevalence of dyslipidemia.

## METHODS

### Study population

Two-stage cluster random sampling was used to cover all 16 states of Palau. First, 75 enumeration areas from among all 130 enumeration areas were selected as the primary sampling units, then even numbers of households were chosen from 75 enumeration areas; thereby, 2,807 households were randomly selected from a total of 3,976 households as the secondary sampling units.^4^ Using the Kish method, one resident aged 25–64 years within each household was recruited for the survey.^3^^,^^4^ From the 2,807 selected households, 2,226 individuals participated in the survey, and available data for 2,184 (1,046 men and 1,138 women) were applicable for the present analyses. Those not within the age range of 25–64 years, who did not indicate sex, and/or women who were pregnant at the time of the survey were excluded.

Palau was under Japanese rule from 1920 to 1945, then controlled by the United States from 1945 to 1994. Therefore, the Japanese and Americans likely influenced Palauan lifestyles. We compared Palau residents with those of Japan and the United States without statistical testing because the data of Japanese and Americans were obtained from published data.^10^^,^^11^ The results from Palau residents aged 30–59 years were compared with Japanese data for corresponding ages, as shown in the National Health and Nutrition Examination Survey 2011, conducted by the Ministry of Health, Labour and Welfare (MHLW) of Japan,^10^ and the data for Americans of corresponding ages were from the United States National Health and Nutrition Examination Survey (NHANES) 2011–2012.^11^ The subjects in these studies were residents sampled using multi-stage probability designs. Japanese subjects totaled 1,428 (550 men and 878 women), and American subjects totaled 2,749 (1,344 men and 1,405 women).

### Data collection

The Ministry of Health conducted STEPS from late 2011 to mid-2013. The STEPS instrument includes three different phases of risk factor assessment. Step 1 is a face-to-face interview based on a structured questionnaire that encompasses age, ethnicity, residential area, education status, marital status, socio-economic information, dietary habits, tobacco and betel nut use, alcohol consumption, and physical activity. For accommodating the specific culture and needs in Palau, this survey was slightly modified from the original STEPS questionnaire. For example, because of common use of betel nuts in Palau, questions about betel nut chewing with or without tobacco were added. Step 2 is physical measurements, including height, weight, abdominal and hip circumference, and blood pressure. Step 3 is biochemical measurements performed in the morning after 10–12 hours fasting. Capillary whole-blood samples were collected using a portable measuring instrument (Accutrend Plus system; Roche Diagnostics, Indianapolis, IN, USA) and total cholesterol and triglycerides were measured.

### Statistical analysis

Dyslipidemia was categorized using the Third Report of the National Cholesterol Education Program Expert Panel on Detection, Evaluation, and Treatment of High Blood Cholesterol in Adults (NCEP ATP III).^12^ Hypercholesterolemia was defined as blood total cholesterol ≥200 mg/dL, and hypertriglyceridemia was defined as fasting triglycerides ≥150 mg/dL. Body mass index (BMI) was calculated as weight in kilograms, measured while wearing light clothes, divided by the square of height in meters, measured barefoot. Overweight was defined as BMI ≥25 kg/m^2^, and obesity was defined as BMI ≥30 kg/m^2^.^13^ Regarding age groups, 18% of residents were aged 25–34 years, 28% were aged 35–44 years, 32% were aged 45–54 years, and 23% were aged 55–64 years. Regarding residential areas, 63% of the population lived in Koror, the main and most populous commercial center of Palau, and 16% in Airai, the second most populous state in Palau, with 21% in other states. In the present study, we defined Koror and Airai as the urban areas and other states as rural areas.^14^ Ethnicity was divided into Palauans and non-Palauans (mostly Filipinos) living in the country. Education was divided into three groups: low education was defined as primary school completion or less, medium as senior high school completion, and high as 2-year college completion or receiving university diploma abroad or higher.

All statistical analyses were conducted using SAS 9.4 statistical software (SAS Institute, Inc., Cary, NC, USA). Tests for linear trend for mean of blood lipids, BMI, and prevalence of hyperlipidemia and overweight/obesity across the age groups were performed by applying the median age and were analyzed as a numeric variable. Multivariable-adjusted mean and prevalence were estimated using multiple regression analysis. Differences in sex-specific and age-adjusted mean values among subgroups of ethnicity, area, and education levels were tested using analysis of covariance. Differences in sex-specific and age-adjusted prevalence of dyslipidemia, overweight/obesity among subgroups of ethnicity, area, and education levels were tested using a chi-square test. All *P*-values for statistical tests were two-tailed and the significance was assessed at *P* < 0.05.

### Ethical considerations

The WHO and the Institutional Review Board of the Ministry of Health, Republic of Palau reviewed and approved the survey. Written informed consent was obtained from all of the participants. The Bioethics Review Committee of Nagoya University School of Medicine approved the relevant research project, including analyses of the STEPS data, in July 2012 (approval no. 2012-0103).

## RESULTS

The mean age of the participants was 45.1 years in men and 45.7 in women. As shown in Table 1, age-adjusted mean BMI was 29 (standard deviation [SD], 6) kg/m^2^ in men and 30 (SD, 7) kg/m^2^ in women (*P* for difference = 0.046). Mean age-adjusted blood triglycerides was 182 (SD, 110) mg/dL in men and 166 (SD, 99) mg/dL in women (*P* for difference = 0.001), and that of blood total cholesterol was 178 (SD, 25) mg/dL in men and 183 (SD, 30) mg/dL in women (*P* for difference = 0.007). The age-adjusted prevalence of overweight/obesity was 75% in men and 76% in women (*P* for difference = 0.84), the prevalence of obesity was 40% in men and 46% in women (*P* for difference = 0.02), the prevalence of hypertriglyceridemia was 48% in men and 41% in women (*P* for difference = 0.002), and the prevalence of hypercholesterolemia was 18% in men and 23% in women (*P* for difference = 0.04). The prevalence of habitual alcohol drinkers who had consumed alcohol 8 or more days during the previous month was 8% in men and 3% in women (*P* for difference <0.001). Betel nut is mostly chewed together with tobacco in Palau. The prevalence of current betel nut chewing with tobacco was 43% in men and 54% in women (*P* for difference <0.001). The prevalence of meals eaten not prepared at home once or more per week was 58% in men and 57% in women (*P* for difference = 0.77). The prevalence of eating fruit ≥3 days per week was 38% in men and 49% in women (*P* for difference <0.001), and that for eating vegetable ≥3 days per week was 71% in men and 81% in women (*P* for difference <0.001).

**Table 1.** Sex- and age-specific means of blood lipids and body mass index, and prevalence of dyslipidemia and overweight/obesity, regular alcohol consumption, tobacco use, and betel nut use in Palau, 2011–2013

Age group, years	Men					

	Total	25–34	35–44	45–54	55–64	*P* for trend
Number of subjects	1,046	193	295	318	240	
Body mass index, kg/m^2^ (SD)	29.3 (6.1)	28.4 (6.6)	29.5 (6.3)	29.6 (5.9)	29.6 (5.8)	0.17
Overweight and/or obesity, %	75	65	75	77	82	<0.001
Obesity, %	40	33	42	42	43	0.13
Triglycerides, mg/dL (SD)	182 (110)	163 (98)	188 (119)	177 (100)	193 (116)	0.04
Hypertriglyceridemia, %	48	41	47	51	52	0.18
Total cholesterol, mg/dL (SD)	178 (25)	173 (22)	177 (25)	178 (26)	184 (27)	0.003
Hypercholesterolemia, %	18	13	17	18	24	0.12
Alcohol consumption >8 days last month, %	8	8	11	7	8	0.49
Current betel nut chewing with tobacco, %	43	45	50	42	36	0.01
Tobacco use only, %	13	17	14	11	12	0.23
Betel nut use only, %	8	3	5	10	14	<0.001
Eating out ≥1 times per week, %	58	68	62	57	45	<0.001
Eating fruit ≥3 times per week, %	38	38	37	39	40	0.95
Eating vegetable ≥3 times per week, %	71	75	75	68	65	0.04

Age group, years	Women					

	Total	25–34	35–44	45–54	55–64	*P* for trend

Number of subjects	1,138	188	312	379	259	
Body mass index, kg/m^2^ (SD)	29.9 (6.6)	28.7 (7.5)	30.0 (7.1)	30.2 (6.1)	30.2 (5.9)	0.06
Overweight and/or obesity, %	76	63	74	80	83	<0.001
Obesity, %	46	39	46	47	47	0.29
Triglycerides, mg/dL (SD)	166 (99)	149 (86)	154 (98)	177 (105)	176 (98)	0.002
Hypertriglyceridemia, %	41	34	34	45	49	0.001
Total cholesterol, mg/dL (SD)	183 (30)	173 (23)	174 (24)	187 (31)	192 (33)	<0.001
Hypercholesterolemia, %	23	14	11	27	37	<0.001
Alcohol consumption >8 days last month, %	3	6	2	3	2	0.02
Current betel nut chewing with tobacco, %	54	58	57	53	47	0.04
Tobacco use only, %	3	5	4	3	2	0.38
Betel nut use only, %	6	2	4	8	11	<0.001
Eating out ≥1 times per week, %	57	66	61	54	48	<0.001
Eating fruit ≥3 times per week, %	49	40	46	53	54	0.003
Eating vegetable ≥3 times per week, %	81	81	80	83	79	0.70

SD, standard deviation.

Sex- and age-specific mean values of total cholesterol tended to be higher with age in men and women. Compared with men, women had the similar mean values of total cholesterol at ages of 25–34 and 35–44 years, but higher mean values in older ages. Mean values of total cholesterol and triglycerides tended to be higher in both older men and women (*P* for trend <0.05). As age increased, the prevalence of hypercholesterolemia and hypertriglyceridemia became higher in women, and those of overweight became higher in both men and women.

**Figure 1.**
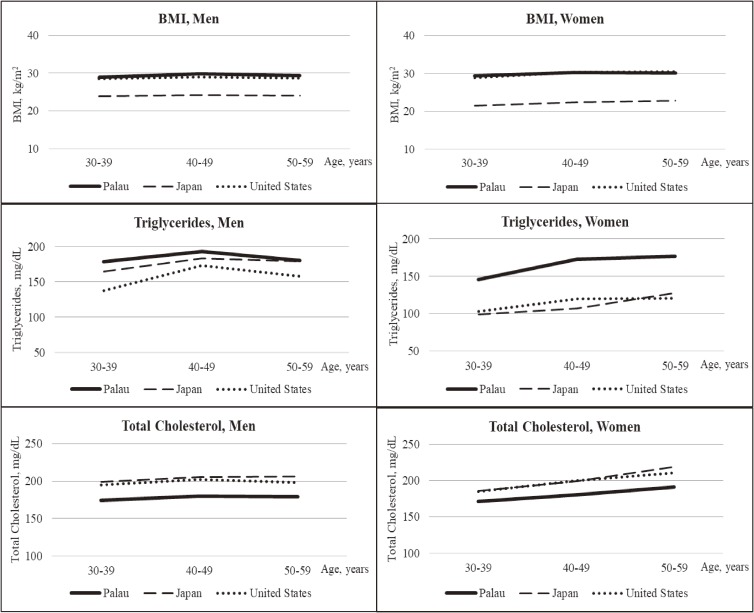
Sex- and age-specific mean values of body mass index and blood lipids in Palauans compared with Japanese and Americans—Palau, 2011–2013; Japan, 2011; United States, 2011–2012

As shown in Figure 1 and eTable 1, mean BMI was higher in Palauans and Americans than in Japanese across the three age groups of 30–39, 40–49, and 50–59 years. Mean values of total cholesterol were lower and those of triglycerides were higher in Palauans than in Japanese and Americans across the three age groups for both men and women.

Table 2 shows sex-specific, multivariable-adjusted mean values of BMI, blood lipids, and prevalence of overweight/obesity and hyperlipidemia according to ethnicity, area, and education level. Regarding ethnicity, 78.7% of participants were Palauans and 21.3% were non-Palauans. Palauan men showed lower mean values of total cholesterol than non-Palauans, but mean values of cholesterol and prevalence of hypercholesterolemia for Palauan women did not differ from that of non-Palauan women. Mean values of triglycerides did not differ between Palauan and non-Palauan men, but were higher in Palauan women than in non-Palauan women, as was the respective prevalence of hypertriglyceridemia in women. Both Palauan men and women had higher respective mean BMI and higher prevalence of overweight and obesity than their non-Palauans counterparts. Adjusting for age, ethnicity, and educational background, mean values of BMI, cholesterol, and triglycerides and prevalence of overweight/obesity and hypertriglyceridemia did not differ between urban and rural areas in both men and women. The prevalence of hypercholesterolemia was higher in rural areas than in urban areas for women, but did not differ for men. After adjusting for age, ethnicity, and area, mean values of cholesterol and triglycerides did not differ by educational background in both men and women. The mean BMI did not vary based on educational background, while higher-educated people showed higher prevalence of being overweight in both the sexes.

**Table 2.** Adjusted mean values of blood lipids and body mass index, and prevalence of dyslipidemia and overweight/obesity according to ethnicity, area, and education level, 2011–2013^a^

	Men				Women			
	
	Ethnicity			*P*	Ethnicity			*P*
	
	Palauans		Non-Palauans		Palauans		Non-Palauans	
Number of subjects	751		293		875		262	
Age, years (SD)	47 (10)		41 (10)	<0.001	46 (10)		43 (10)	<0.001
Body mass index, kg/m^2^ (SE)	30.8 (0.2)		25.4 (0.4)	<0.001	31.3 (0.2)		25.2 (0.4)	<0.001
Overweight and/or obesity, %	84		53	<0.001	86		45	<0.001
Obesity, %	52		12	<0.001	55		15	<0.001
Triglycerides, mg/dL (SE)	182 (4)		179 (7)	0.68	171 (4)		150 (7)	0.006
Hypertriglyceridemia, %	49		48	0.86	44		34	0.007
Total cholesterol, mg/dL (SE)	177 (1)		182 (2)	0.03	182 (1)		186 (2)	0.09
Hypercholesterolemia, %	16		24	0.02	23		24	0.64

	Area			*P*	Area			*P*
	
	Urban		Rural		Urban		Rural	

Number of subjects	802		244		917		221	
Age, years (SD)	44 (10)		47 (10)	<0.001	46 (10)		46 (10)	0.64
Body mass index, kg/m^2^ (SE)	29.4 (0.2)		29.1 (0.4)	0.45	29.9 (0.2)		29.9 (0.4)	0.94
Overweight and/or obesity, %	77		72	0.10	76		77	0.77
Obesity, %	40		41	0.84	46		43	0.30
Triglycerides, mg/dL (SE)	185 (4)		173 (7)	0.18	166 (4)		167 (7)	0.90
Hypertriglyceridemia, %	49		46	0.49	40		46	0.19
Total cholesterol, mg/dL (SE)	179 (1)		176 (2)	0.20	182 (1)		187 (2)	0.049
Hypercholesterolemia, %	19		16	0.39	21		32	0.008

	Education			*P*	Education			*P*
	
	Low	Medium	High		Low	Medium	High	

Number of subjects	183	428	425		164	432	534	
Age, years (SD)	45 (10)	45 (10)	45 (10)	0.77	48 (11)	46 (10)	45 (10)	0.008
Body mass index, kg/m^2^ (SE)	28.5 (0.4)	29.5 (0.3)	29.6 (0.3)	0.08	29.7 (0.5)	30.3 (0.3)	29.6 (0.3)	0.16
Overweight and/or obesity, %	69	77	77	0.05	73	76	80	0.02
Obesity, %	35	41	42	0.17	43	48	50	0.11
Triglycerides, mg/dL (SE)	177 (9)	179 (6)	186 (6)	0.57	161 (8)	166 (5)	168 (5)	0.73
Hypertriglyceridemia, %	45	48	50	0.64	36	43	41	0.34
Total cholesterol, mg/dL (SE)	181 (3)	179 (1)	177 (1)	0.39	182 (3)	185 (2)	182 (2)	0.38
Hypercholesterolemia, %	23	19	16	0.21	22	24	23	0.87

SD, standard deviation. SE, standard error.

^a^Adjust model include age, ethnicity, area, and education.

## DISCUSSION

In the present study of adults (men and women combined) aged 25–64 years in Palau, mean value of BMI was 30 (SD, 6) kg/m^2^, mean triglycerides value was 173 (SD, 104) mg/dL, and mean total cholesterol was 181 (SD, 28) mg/dL. Seventy-six percent of adults aged 25–64 years of Palau were overweight/obese (BMI ≥25 kg/m^2^), 43% were obese (BMI ≥30 kg/m^2^), and 45% had hypertriglyceridemia (triglycerides ≥150 mg/dL). However, only 21% had hypercholesterolemia (total cholesterol ≥200 mg/dL).

Traditional Palauan foods, such as taro, fish, coconut, and various fruits, were widely consumed in Palau in the early 1900s.^2^^,^^15^ These foods comprised the core of low-fat, low-calorie, high-fiber diets. The mean BMI in Palau was reported as 24 kg/m^2^ in the 1950s^16^ but had reached nearly 30 kg/m^2^ as of the present study. Palau has also been reported as having the seventh highest mean BMI in the world as of 2007.^16^ This is because substantial dietary changes have occurred in Palau over the past half-century, with declining reliance on traditional foods and increased use of imported and processed foods. White rice replaced taro, processed meats or canned fish replaced local fresh seafood, and processed snacks and sweetened beverages replaced local fresh fruits.^17^ Additionally, many Palauans are clerical workers employed in the public sector, use cars for their main form of transportation, and have domestic helpers for housework.^18^^,^^19^ As a result, daily physical activity of the country’s people has decreased. Eating too much rice and canned and processed foods, low intake of fresh vegetables and fruit, and insufficient physical activity are considered main factors of the high prevalence of obesity^20^^,^^21^ and hypertriglyceridemia.^22^ The high prevalence of obesity is said to originate from norms about body shape among people living in Western Pacific countries: many Palauans still believe high weight is a symbol of health, wellbeing, and beauty.^23^

As Palauans have such lifestyles, along with high prevalence of overweight/obesity and hypertriglyceridemia, they can be expected to have high prevalence of hypercholesterolemia. However, we found they showed low mean cholesterol values. eTable 2 shows mean values of total cholesterol, triglycerides, and BMI, and prevalence of their elevated values in Pacific Island countries under the WHO STEPS.^24^ Most Pacific Island countries showed a lower mean value of cholesterol and lower prevalence of elevated cholesterol compared with figures according to data for Japanese and Americans, and Palau also showed the same trend as other Pacific Island countries. These findings suggest that low total cholesterol levels in Palauans may be due to genetic factors rather than environmental and behavioral factors.

In the present study, women also showed a higher mean BMI than men. According to STEPS reports on Pacific Island countries, women have historically shown a higher proportion of overweight/obesity than men. This is a similar observation as in a previous review, in which five of six WHO-categorized regions (Africa, Americas, Eastern Mediterranean, South-East Asia, and Western Pacific), with the exception of Europe, showed higher BMI among women than among men.^25^

Large differences were found in the prevalence of overweight/obesity, hypertriglyceridemia, and hypercholesterolemia between Palauan and non-Palauan residents of Palau. Most non-Palauans are migrant workers, and most such workers are in low-paying fishing, domestic help, and production and construction jobs, while most Palauans are clerical workers.^18^^,^^19^ The low prevalence of hypertriglyceridemia, overweight and obesity among non-Palauans compared with Palauans may be attributable to greater physical labor and inferior work conditions. Another reason for this preferable profile of blood lipid in non-Palauans is the healthy worker effect. Non-Palauan workers are obligated to undergo an annual health checkup, and when certain health problems are found, they must be repatriated to their home countries.

The present study identified no difference among rural and urban areas in triglycerides and BMI but did find a difference in the prevalence of hypercholesterolemia for women. There were no differences in the prevalence of dyslipidemia by educational level, but higher-educated people had a higher proportion of being overweight among both men and women. Education is typically viewed as an indicator of socioeconomic status.^26^ Higher-educated people are more likely to come from higher-income families and have a greater chance to be government employees and to be engaged in sedentary work. High socioeconomic status and lack of physical activity are probably the main reasons for the positive correlation between educational level and being overweight. A systematic review of 289 articles that reported on 410 populations in 91 countries found higher education was associated with higher prevalence of obesity in developing countries, while the opposite trend was observed in developed countries.^27^

The present survey had several limitations. First, we did not examine HDL- and LDL-cholesterol levels; therefore, we may have failed to acquire a more precise picture of lipid profiles. Second, we did not obtain detailed information on consumption of food, soft drinks, and alcohol to help explain the profiles of blood lipids and BMI. Third, we measured triglycerides and cholesterol levels with portable instruments, while the data on Japanese and Americans were measured using laboratory-based methods. It has been reported that triglyceride levels measured using portable instruments are about 9% higher than those using laboratory-based methods with venous blood samples, while only a 1.4% difference was found for cholesterol levels.^28^ Even when taking the overestimated levels into account, mean triglycerides were still higher in Palauan women than in American and Japanese women. Last, we tested multiple hypotheses, so some of the associations could be due to chance.

This is, to our knowledge, the first population-based survey on metabolic risk factors of NCDs among adults in Palau. The present study revealed a high prevalence of overweight/obesity and hypertriglyceridemia but a low prevalence of hypercholesterolemia in Palau. Compared with non-Palauans, Palauan men and women both had higher mean BMI and higher prevalence of overweight/obesity, and Palauan women had higher mean triglycerides and higher prevalence of hypertriglyceridemia than non-Palauan women. Adults aged 25–64 years in Palau with higher education were more likely to be overweight, which may permit room in which health education interventions could be feasible and effective in the prevention and control of overweight and obesity.

## APPENDIX A. SUPPLEMENTARY DATA

eTable 1. Sex- and age-specific mean values (standard deviations) of body mass index and blood lipids in Palauans, Japanese, and Americans—Palau, 2011–2013; Japan, 2011; and United States, 2011–2012

eTable 2. Sex-specific mean values of body mass index, blood lipids, and prevalence of overweight/obesity and dyslipidemia in Pacific island countries and territories according to WHO STEPwise approach to Surveillance country report
