# Supplementary material for: Prevalence and Correlates of Dyslipidemia Among Men and Women in Palau: Findings of the Palau STEPS Survey 2011–2013
Source: J Epidemiol. 2019 Mar 5;29(3):97–103. doi: 10.2188/jea.JE20170127 (PMC6375813; doi:10.2188/jea.JE20170127)
Supplement: Supplementary file 1 [file je-29-097-s001.pdf]

eTable 1. Sex- and age-specific mean values (standard deviations) of body mass index and blood lipids in Palauans, Japanese, and Americans — Palau, 2011–2013; Japan, 2011; and United States, 2011–2012

	Men			Women		
Age group, years	30–39	40–49	50–59	30–39	40–49	50–59
**Palau**						
Number of subjects	239	332	292	247	379	315
Body mass index, kg/m^2	28.9 (6.3)	29.8 (6.2)	29.4 (5.9)	29.3 (6.8)	30.3 (6.6)	30.1 (5.9)
Triglycerides, mg/dL	179 (109)	193 (118)	179 (105)	146 (81)	173 (111)	177 (102)
Total cholesterol, mg/dL	174 (23)	180 (25)	179 (26)	171 (19)	180 (26)	191 (33)
**Japan**						
Number of subjects	181	154	215	273	277	328
Body mass index, kg/m^2	23.8 (3.9)	24.2 (3.7)	24.0 (3.3)	21.5 (3.4)	22.4 (3.5)	22.8 (3.7)
Triglycerides, mg/dL	165 (125)	183 (130)	179 (132)	99 (64)	107 (82)	128 (77)
Total cholesterol, mg/dL	199 (35)	205 (32)	206 (33)	186 (33)	199 (31)	219 (33)
**US**						
Number of subjects	481	428	435	459	468	478
Body mass index, kg/m^2	28.5 (6.4)	29.0 (6.3)	28.6 (6.0)	28.8 (7.7)	30.4 (8.2)	30.5 (7.5)
Triglycerides, mg/dL	138 (101)	173 (120)	158 (147)	103 (62)	120 (126)	121 (71)
Total cholesterol, mg/dL	195 (42)	202 (39)	198 (43)	185 (34)	200 (37)	211 (44)

SD, standard deviation.

Source: Japanese data based on National Health and Nutrition Examination Survey 2011; Americans data based on National Health and Nutrition Examination Survey 2011–2012.

eTable 2. Sex-specific mean values of body mass index, blood lipids, and prevalence of overweight/obesity and dyslipidemia in Pacific island countries and territories according to WHO STEPwise approach to Surveillance country report

Country	Year	Body mass index, kg/m^2		Overweight and/or obesity,%[a]		Total cholesterol, mg/dL		Cholesterol ≥190 mg/dL, %		Cholesterole ≥200 mg/dL, %		Triglycerides, mg/dL		Triglyceridemia ≥150 mg/dL, %	
		Men	Women	Men	Women	Men	Women	Men	Women	Men	Women	Men	Women	Men	Women
Palau	2013	29.3	29.9	75.5	76.1	178.3	182.8	26.0	33.3	18.2	23.3	181.4	165.7	48.3	41.2
American Samoa	2004	33.7	36.2	92.7	94.4	181.7	185.6			23.4	23.7				
Cook Islands	2004	32.3	33.3	89.8	87.1	197.2	190.0	77.1	73.2						
Fiji	2002	24.2	26.7	37.3	57.9	208.4	194.1			49.1	37.8	130.2	116.0	29.6	23.3
French Polynesia	2010	29.1	29.4	71.1	68.7	190.6	187.8	44.0	43.5						
FSM (Chuuk)	2006	28.2	32.7	65.0	88.0	175.0	183.0	12.0	24.0						
FSM (Pohnpei)	2002	27.7	31.1	64.0	83.0	200.0	200.0			48.4	45.0				
Kiribati	2006	29.4	31.5	78.2	84.6	176.6	183.2	23.8	30.6						
Marshall Islands	2002	26.7	28.5	59.9	65.5	165.5	173.8			20.3	22.9	103.5	92.9	9.5	6.0
Nauru	2004	31.7	32.5	82.1	82.2	166.3	174.0			14.9	20.8				
Niue	2011	31.1	32.5	85.0	86.8	177.3	179.1	33.7	36.0						
Samoa	2002	30.0	33.2	81.1	89.8	162.8	163.9			13.8	13.6				
Solomon Islands	2006	27.2	29.0	62.5	72.7	174.7	181.3	19.6	28.5			136.7	140.0	35.0	30.9
Tokelau	2005	31.5	33.2	82.9	89.3	189.5	190.0			33.8	37.0				
Tonga	2004	31.7	34.9	89.2	94.9	208.6	184.4	66.1	34.2			154.6	123.9	36.7	34.9
Vanuatu	2011	25.5	26.7	45.5	55.9	192.8	188.6	38.4	34.7						

FSM, Federated States of Micronesia.

[a] Overweight/Obesity was defined as BMI≥$25kg/m^2$.
